# Between Emotional Involvement and Professional Detachment: The Challenges of Nursing in Dutch Mental Institutions (1880–1980)

**DOI:** 10.1093/shm/hkaa086

**Published:** 2021-02-19

**Authors:** Harry Oosterhuis, Cecile Aan de Stegge

**Affiliations:** 1 Faculty of Arts and Social Sciences, Maastricht University, PO Box 616, Maastricht, 6200 MD, The Netherlands; 2 Nursing Department of the Faculty of Health, Leyden University of Applied Sciences, the Netherlands; 3 teaches history at the Faculty of Arts and Social Sciences of Maastricht University, Maastricht, The Netherlands; 4 lecturer in the Nursing Department of the Faculty of Health, Leiden University of Applied Sciences, Leiden, The Netherlands

**Keywords:** Dutch psychiatry, mental nursing, emotional work, coercion, sexuality, suicide

## Abstract

This article is about the tension and changing balance between emotional involvement and professional detachment in the practice of nursing in Dutch mental institutions between the 1880s and 1990s. We address this issue in relation to institutional and material conditions, power differences between doctors, nurses and patients, different treatments, and the social marginalisation of hospitalised patients. On the basis of various sources (nursing textbooks, chronicles of skills learning by students, personal accounts, questionnaires and interviews), we describe how nurses were supposed to interact with patients and how they dealt with three sensitive issues: the need to use coercion in response to agitated patients, the sexual behaviour of patients and the risk of suicide in psychiatric institutions. We argue that nursing mental patients required a great deal of emotional work and that there was a shift from strict rules of behaviour imposed from above to more flexible self-regulation, guided by self-reflection.

In 1916 and 1917, the *Monthly Journal of the Dutch Labour Union for Nurses* published a series of six articles about mental nursing by Henri van den Bor (1885–1953), who worked in this field and played a key role in the union. He noted a high turnover among nurses in asylums and a shortage of capable staff. Experienced nurses had to show the ropes to new apprentices over and over again. For many youngsters, the work was far more difficult than expected, and they barely realised many daily challenges involved. The treatment of the insane, according to Van den Bor, required a particular attitude and commitment. Nurses were expected to deal with the ‘peculiarities’, ‘irritability’ and ‘most bizarre habits’ of patients, but also to approach them as much as possible as normal people in a polite way.^1^ Apart from their varying mental symptoms, nurses should have consideration for their social class, self-esteem and worldview. Conversations about political and religious issues had to be avoided. Nurses should be aware of the drastic impact on patients of their admission to an asylum, where they were separated from their family and deprived of their legal status, control over their income and property, and their usual social ties. Dealing with differences in character, intelligence or energy, while still treating patients as equals, was another requirement of mental nursing. Understanding patients' emotions—sorrow, shame, loneliness, feeling offended and aggression—was of the utmost importance. Nurses themselves should always remain calm, cautious, patient and in control. If they had to use coercion, inflicting pain had to be avoided and they should prevent patients from feeling imprisoned. Nurses should be prepared for possible suicides and supervise patients at risk conscientiously but imperceptibly. Homosexual contacts between patients—men and women were strictly segregated—had to be countered, without confronting them directly with suspicion in order to prevent needless offence. Nurses were allowed to show their interest if patients wanted to share their ‘secrets’, but they should not provoke such intimacies.^2^ If patients asked about their illness or medical treatment, they should be referred to the doctor, whose wisdom nurses should never question. Empathy and communication were essential requirements for mental nursing, but such engagement should not lead to being overwhelmed by the suffering they faced on a daily base. Like a surgeon would be unqualified for his work if each incision caused him to be overwhelmed by compassion, so nurses would not perform well without keeping a certain measure of distance to patients, even though this should never result in indifference on the part of nurses. In other words, finding a proper balance between involvement and detachment was of utmost importance. 

## Professional Psychiatric Nursing and its Challenges

The views presented by Van den Bor implicitly reveal a kind of insight that more recently has been discussed theoretically in sociological and historical studies on professionalisation: the importance of practical and intuitive skills. Occupations are often defined on the basis of formal and institutional characteristics: licenced training schemes, examinations and diplomas; codes of behaviour, sense of duty and public service; internal solidarity and control over competences; and boundary work vis-à-vis other professions. But if we want to know what mental nursing—or any other occupation—is really about, informal practices and experiences, usually acquired on the shop floor in an improvisatory, pragmatic and intuitive way, are at least as relevant.^3^ Van den Bor’s specific recommendations, formulated in the 1910s, were a sign of the times, but the more general issue he raised still touches on the core of help-oriented professions in general and nursing in particular. These occupations deal with dependent and suffering people in need of care, and the way patients or clients are treated is a significant benchmark for the quality of the work. Merely applying professional knowhow in an instrumental and standardised manner, without empathy, is likely to give rise to dissatisfaction, if it is not experienced as hurtful. Conversely, some distance is necessary: excessive emotional involvement is at odds with the rationality and objectivity of professionalism. Professional care should not only meet the requirements of impartiality, efficiency and scientific validation, but also be in line with democratic values, such as respect for personal dignity and autonomy, equal treatment and distributive justice.^4^ Arbitrariness and misuse of power should be ruled out. In the welfare state, the financial costs and the regulation of health care have become a collective responsibility, causing it increasingly to be relegated to professionals, who have to handle its emotional aspects on behalf of the public at large.

Finding and keeping a balance between involvement and detachment requires considerable ‘emotional labour’.^5^ In psychiatry such emotional labour is complicated because the mentally ill do not fit in the normality and self-evidence of regular social life. The essence of mental illness concerns behaviours and feelings which are generally viewed as weird, erratic, painful or even dangerous, and which are often shunned and stigmatised. As Van den Bor noted, interacting with psychiatric patients is not easy; this is why he stressed that mental nursing called for specific character features and emotional skills. Moreover, the relation between nurses and patients tends to be put to the test because of the need to impose unpleasant measures, such as several forms of constraint. In the past as well as today, the dilemma of compassion and detachment goes to the heart of mental nursing. This does not imply, however, that the specific quality and the interrelationship of the associated attitudes remained the same. Daily interactions between nurses and patients change over time, as is true of norms and ideals articulated in training programmes and formal rules of professional behaviour.

This article is about changing nursing practices between the 1880s and 1980s against the background of developments in Dutch psychiatric institutions: their material provisions, institutional structures and rules; relationships of authority and power between doctors, nurses and patients; medical and psychosocial treatments; and the judicial dimension of hospitalisation. Which ideals, rules and recommendations guided the way nurses should associate with patients? Who defined them and what was the relation between norms and realities? How did nurses deal with the tension between involvement and detachment and how did their interaction with patients change in the course of time? This last question is the leading one in our analysis of the manners between nurses and patients. We focus on three sensitive issues highlighted by Van den Bor: aggression and violence, sexuality and suicide. In part, because of the assumed lack of self-control among the mentally ill, the aim of constraining these inclinations and behaviours played a prominent role in both the specific literature and the everyday practice of nursing. Our study is based on five primary sources: (1) textbooks for mental nursing training programmes; (2) report booklets recording and evaluating the skills of student-nurses^6^; (3) accounts by nurses about their experiences in daily work, in particular the essays which 84 of them wrote on the occasion of a writing contest organised in 1941^7^; (4) a questionnaire completed by more than one hundred nurses about their views of their training and daily work^8^; and (5) several interviews with nurses.^9^ Based on the changes we perceive in both the expected and the actual attitude of nurses vis-à-vis patients—overall a shift from imposed and strict rules of conduct to more flexible forms of self-regulation—we distinguish four partly succeeding and partly overlapping ‘regimes of care’: a regime of ‘subservience’ (late nineteenth century until late 1920s), a regime of ‘self-responsibility’ (from the 1930s onwards), one of ‘personal development’ (from the 1950s onwards) and one of ‘emancipation’ (from the late 1960s onwards).

## The Regime of Subservience

Until the late nineteenth century, the presence of physicians in lunatic asylums was limited and nursing care was rare. The main task of the attendants and, in Catholic institutions, religious brothers or sisters involved the control of the inmates’ troublesome behaviour. Coercion, physical restraints and solitary confinement were common. Low salaries and poor working and living conditions caused a high staff turnover. Maltreatment of patients by attendants added to the social stigma of asylums, the more so because some former patients complained about them in public.^10^ State supervisors and doctors who advocated a humane and medical approach repeatedly condemned the incompetent and ‘uncivilised’ attendants. The insane should be nursed, they argued, on the basis of hygienic guidelines, middle-class standards of proper behaviour, and ethical and religious values, such as compassion, patience and helpfulness.^11^ From the 1870s onwards, doctors increasingly established control over the management of asylums. Their aspiration to transform them into medical facilities through replacing the unqualified attendants by motivated and trained nurses was boosted by the Dutch Association of Psychiatry and Neurology, founded in 1871, and the Insanity Law adopted in 1884. This law not only introduced standards for medical care in asylums, the keeping of patient records and the use of coercion against patients, but also referred to the need for better attending staff. In the 1880s and 1890s, several new mental institutions were built, all situated in the countryside and equipped with medical facilities.^12^

In the 1880s and 1890s, nurse training by senior doctors was introduced in more and more asylums and formalised in a three-year curriculum. To a large extent, mental nursing followed the example of nursing in general hospitals, which was modelled on prevailing ideas about the division of gender roles. Male doctors defined the required knowledge, skills and attitudes, which were tailored to ‘civilised’ middle class women. Leading asylum doctors stressed that psychiatric nursing, like its somatic counterpart, was primarily an occupation for women, because of, as one of them put it, their ‘sensitivity, tact, helpfulness’ and ‘civilising and educational talent’.^13^ Nursing care had to be subservient, in line with the assumed caring nature of women. Physicians considered scientific knowledge and competence to be irreconcilable with emotional involvement. It was up to nurses to provide sensitive care, and accordingly, they began to function as a kind of soft buffer between physicians and patients. Since the late nineteenth century, nurses constituted by far the largest group among the staff in mental institutions, but their voices could be heard only sporadically: their work largely remained invisible—just as running a household by housewives was taken for granted and often undervalued.^14^

In most western countries, mental nursing developed as a specialisation within a single general training scheme for all nurses, but in The Netherlands, this occupation, under full control of asylum doctors, was set up as a distinct branch, separately from somatic nursing. Apart from basic medical skills, instructors stressed social and didactic abilities, while in Protestant and Catholic asylums, moral and religious values were emphasised as well.^15^ Mental nursing also distinguished itself with regard to class and gender. The first efforts by asylum doctors to enlist middle class women with a secondary education largely failed because they generally preferred working in a general hospital: the stigma associated with the insane, their disruptive conduct, the poor wages and the mandatory internship in remote asylums discouraged these women from opting for psychiatric nursing. Apart from the religious staff in Catholic institutions, asylums largely depended on young women, and also men, from the lower middle and working classes, most of whom only enjoyed primary education. Doctors took pains to remedy their lack of education by offering them extra training, while also claiming that the background of these nurses could be advantageous: most patients also came from the lower classes and would feel more at ease with nurses with a similar background. Moral calibre and character traits such as calmness, patience and tact were more useful, doctors contended, than intellectual education or bourgeois attitudes.^16^ Moreover, the need to handle the unruly behaviour of male patients and the economic and therapeutic importance of work entailed that also men, in particular those skilled in crafts, were hired as nurses. Throughout the twentieth century, their share fluctuated between 30 and 40 per cent of the total nursing staff in Dutch mental institutions, while in general hospitals, the percentage varied from 3 to 10 at the very most.^17^ Because of their gender and distinct skills, the subservience of male nurses to doctors was not as strong as that of their female colleagues. Some of these nurses, such as Van den Bor, reflected on the social and didactic orientation of psychiatric nursing and played a central role in the first labour unions for nurses.

All the same, until the 1960s male and female nurses themselves had little say about the contents and organisation of their training and work. The hierarchy of academically educated, bourgeois physicians versus lower class nurses was part of the largely authoritarian and rigid organisation of asylums, in which coercion was pervasive. Asylums were closed institutions, and admission was by definition enforced: until around 1930, virtually all patients were hospitalised on the basis of legal certification, which implied loss of full citizenship. To prevent patients from running away, high partitions or walls separated wards, while nurses constantly had to check the locks on doors and windows. They could be fined, if not fired, when a patient managed to run away due to their inattentiveness.^18^ The asylum life was structured around strict rituals and do’s and don’ts, for patients as well as nursing staff. Insane people were seen as more or less immature people without sufficient self-control, and therefore, they were not accountable for their unpredictable behaviours and expressions. Much like children, they needed intensive external guidance. This approach advanced considerable mental distance between nurses and patients, even though nearly all the time they were physically close to each other. The similar inelegant outfits and haircuts of patients, their rather dreary appearance versus the tidy uniforms of nurses brought about a depersonalising effect, which was strengthened by using family names and addressing each other with the formal version of ‘you’.

At the same time, the nursing textbooks emphasised that the heartless and at times cruel treatment of the insane was a thing of the past and that nurses had to treat the mentally ill in a humane way, regardless of their strange and worrisome expressions. They were expected to protect the mentally ill from neglect and relate to them kindly and patiently. Patients needed daily pursuits and in order to distract their troubled minds nurses should associate with them in their daily life on the wards or in a workplace. Nurses were taught that the disturbed and aggressive behaviours of the mentally ill did not originate in evil intent, but rather were symptoms of their illness, to which they were supposed to respond in a controlled, more or less casual, and even kind-hearted way. A humane treatment would make asylums more ‘sociable’, as several authors of textbooks put it: the ‘hate, grudges and animosity’ of patients vis-à-vis staff would clear the way for ‘kindness and trust’.^19^

The partly contradictory assumption that the mentally ill were like ‘irresponsible children’ but also 'fellow human beings’ was highlighted in particular in the rules which nurses should punctually follow when dealing with disorderly and aggressive behaviour. The aim of asylum doctors to reduce physical coercion in mental hospitals and model these after general hospitals was a major motivation for their design of the nursing curriculum. The guidelines in their textbooks were hardly unequivocal, however. If they discouraged and argued against the use of coercion, the examination regulations also stipulated that nurses had to ensure ‘quietness’ among the patients and this also entailed, if necessary, coerced calmness.^20^ As revealed by the evaluation report booklets, student nurses trained these skills in practice-oriented classes. In fact, the application of coercion was inescapable, and doctors relegated its implementation and the direct responsibility for it to the nurses.^21^

That the use of physical coercion was increasingly seen as painful and less acceptable is suggested by the state inspectors’ demand that the use of mechanical coercive tools or means—forced wearing of straightjackets, tying hands and/or feet with belts while sitting on a chair (‘coercive chair’)—and isolation had to be motivated and recorded.^22^ If this recording may not have always been systematic, physical coercion with mechanical means was increasingly regarded as a sensitive issue calling for limitation. This gave rise to other methods which were seen as less offensive or less terrifying and did not have to be recorded, such as manual restraint, the ‘wrapping’ in dry or wet sheets, and ‘medical’ methods, such as ‘bedside nursing’, prolonged ‘bath therapy’ or tranquilising drugs.^23^ The former ‘isolation cells’ without windows and furniture and with heavily locked doors in a separate and remote section (reminiscent of prison cells) in which patients were secluded—and which engendered the feeling of locking up people, like dogs or wild animals, in cages—were increasingly replaced by furnished ‘single rooms’ with a window close to the regular wards, making their isolation less total and more temporary.^24^ The approach of leaving a ‘wrapped’ patient alone on a ward among the other patients now became an acceptable alternative for isolation.^25^ In addition, so-called ‘monitoring rooms’ were put in near the sleeping wards so as to be able to keep an eye on patients who were suicidal or prone to self-mutilation or ‘indecent’ conduct.^26^ This is not to suggest that in a physical or emotional sense these more humane forms of coercion were less burdensome or confrontational for nurses. For example, ‘holding’ an agitated patient whereby nurses used their own body could last for hours and be as labour-intensive as wrapping the patient’s body in a sheet and the subsequent periodic cleaning and feeding of these ‘living mummies’.^27^

Although sexuality was subjected to all sorts of prohibitive rules, which partly were intrinsic to the institution’s strict organisation, this issue was a taboo in the nursing textbooks, unlike coercion and suicide. Yet, sexuality was a source of great tension in closed asylums, where patients lived apart from partners and sexual gratification was virtually made impossible. Until the 1960s, male and female patients were strictly separated, and they were as much as possible cared for by nurses of their own sex. Under no conditions, male nurses were allowed to work on women’s wards, while female nurses were actually deployed on men’s wards. This asymmetry mirrored common views on the relation between sexuality and gender and class. Men would be driven more strongly than women by sexual desires, while those in lower classes would be less capable of suppressing sexual urges. Female patients were deemed vulnerable to sexual enticement or abuse by male nurses—deploying them on women’s wards was seen as equal to ‘leaving the fox to watch the geese’—while female nurses would potentially be in risky situations on men’s wards.^28^ Nurses had to avoid every association with sexuality, in order not to arouse patients in any way.^29^ Long dresses with aprons, black stockings, sturdy shoes and pinned-up hair underlined the sexless appearance of female nurses. They were expected to be alert constantly and everywhere for possible sexual expressions by patients, even when they had to go to the toilet—in covered terms the textbooks referred in particular to masturbation and ‘perverse’ (mainly homosexual) acts—and to make sure that such acts were nipped in the bud. The fear was that every ‘immoral’ act would further extend such behaviour. Such close permanent control applied in particular to patients who were agitated and ‘indecent’, whereby their privacy or feeling of shame, such as when taking a bath or visiting the toilet, was little respected.^30^

The nursing textbooks paid much attention to suicide. Research based on patient files, as well as the recorded experiences of nurses, reveal that the risk of suicide played a large role indeed in asylum life and emotionally burdened the nursing staff. Just as patients disposed to overt sexual behaviour, suicidal patients were closely monitored by nurses.^31^ The monitoring and care of such patients, as one of the textbooks indicated,

“constitutes one of the hardest parts of nursing the insane. Every suicide is unique and has many implications. It is only in rare cases that there is no one to blame at all, because often a lack of caution or proper judgement will be involved at some level, or negligence or inconsiderateness in surveillance and monitoring”.^32^

Nurses were not allowed to leave alone suicidal patients, including those in isolation. Furthermore, they constantly had to check patients on their possession of possible ‘dangerous materials’, such as knives, scissors, pieces of glass, matches, ropes and medicines. After each meal, the cutlery had to be counted, as was true of tools used in occupational therapy. In the case of a suicide (attempt), nurses could be fired if their ‘negligence’ or ‘guilty oversight’ was established.^33^ A case of suicide was normally followed by a judicial inquiry, whereby all involved nurses were interrogated. Nurses carried a lot of responsibility for preventing suicide and estimating the risk of suicide, but in case of an attempt, they were also the ones to provide emergency care, including reanimation.^34^ Having to deal with suicide and take care of the dead body usually took a toll on nurses, while the asylum doctors kept a distance as their involvement was limited to treating wounds or establishing the cause of death. The emphasis on continuous monitoring of suicidal patients and the potentially severe consequences in case of a suicide (attempt) also contributed to an atmosphere of fear, concealment and distrust between nurses and patients, all the more so because nurses, due to the assumed ‘risk of infection’ or ‘contagiousness’ of suicidal conduct, were not allowed to speak openly about suicide.^35^

## The Regime of Self-Responsibility

The first shift from a strict regime of do’s and don’ts to a regime of increased self-responsibility of nurses as well as patients was advanced by the introduction of the so-called (more) active therapy, imported from Germany from 1926 onwards and widely used in Dutch mental institutions well into the 1960s. Occupational therapy, used since the nineteenth century for economic reasons and as part of moral treatment, was transformed into a didactic method for stimulating patients’ social behaviour and accountability. Some leading psychiatrists focussed on the corrective behavioural conditioning of patients, but others, following a psychosocial approach, adopted active therapy for intensifying meaningful interaction between nurses and patients. Not only should nurses work together with patients, but they should also take part in their daily routines, such as communal meals and recreational activities, including sports and creative pursuits.^36^ Much more than before, nurses were expected to empathise with patients, to have an understanding of their daily world and personal preferences and dislikes, and thus win their trust. The new approach was enhanced by changes in the patient population. From around 1930 on, in addition to the existing court-sanctioned admission, uncertified hospitalisation of patients suffering from nervous disorders, psychosomatic complaints or mild psychosis increased.^37^ These patients, staying in open departments of mental institutions, were generally more approachable and communicative, but also uttering more criticism about their treatment and thus more demanding than many of the insane. Nurses took the role of confidential intermediary between these articulate patients and doctors, and this advanced more equal and empathic interactions. More sophisticated observational and communicative skills were added to the training schemes for nurses.^38^

The new approach appealed to nurses and changed their daily work, as can be gathered from the essays which they submitted on the occasion of a writing contest organised by the Dutch Psychiatric and Neurological Association in 1941.^39^ Nurses expressed themselves frankly about their experiences and motives, and some of them showed outspoken ideas on their occupation. Many of them referred to their role in active therapy and reported improvement of the institutional living conditions and, to their own surprise, in the behaviour of many previously apathetic or agitated patients. The need to apply restraints and isolation had dwindled. More personal interaction, in particular through joint recreation, had brought them closer to individual patients.^40^ Through their daily involvement with the mentally ill, nurses began to influence the practice of care in significant ways. The stimulating and partly individualised approach of active therapy put high demands upon nurses because it required continuous attention, vigilance, flexibility and tact. Higher demands also applied to patients: instead of docility more participating behaviour was the goal. Disturbing behaviour should not be merely suppressed, but it had to be transformed into a social attitude—an aspiration that provoked resistance of some patients. Restless patients were briefly isolated and returned sooner to the group (and, if needed, secluded again).^41^ Physical coercion was deemed even less desirable than before. This became clear when from 1930 onwards more methods needed to be recorded, such as the use of straightjackets, prolonged bath therapy and seclusion, while state inspection of these was intensified.^42^ Participation in occupational therapy or other group activities had to curtail mechanical restraints and lengthy isolation as much as possible. The aversion to physical restraint among nurses also increased through the decreasing emotional distance vis-à-vis patients.^43^ As a nurse wrote in her essay, she felt ‘an inward resistance’ when there was an undeniable need to restrain or lock up ‘a fellow man’ and she hoped that in the future ‘such spiritually painful things would not be necessary anymore’.^44^ Another nurse criticised the use of the so-called ‘wet wrapping’ to quiet down patients: he felt it to be a ‘horrible’ method and he did not believe that ‘one could ever calm a person in such a way’.^45^

In the 1930s, mental nurses, of whom a higher educational level was required at their entrance, began to develop some professional self-confidence and more interest in the social, psychological and ethical dimension of their work. This last dimension was highlighted by the author of the prizewinning essay ‘Respect for Life’, which addressed the issue of how to deal emotionally with ‘the hopeless suffering’ witnessed each and every day. It had to be accepted as part of ‘the whole of creation’, a statement that was in fact a criticism of the coercive ‘euthanasia’ of mentally disabled patients in Nazi Germany.^46^ Some nurses also began to express disapproval with respect to drastic somatic treatments such as the malaria fever, electroshock and insulin coma therapy.^47^ One nurse noted in his essay about the latter that it ‘leads us to a dark way of trial and error, with many shady sides. […] The support nurses have to give is far from simple, yet full of responsibility. One single moment of inattention can lead to the patient’s death’.^48^ Another nurse criticised somatic therapies because they reintroduced bed rest, which—like in the old days—had made patients ‘passive and weak’.^49^

The introduction of more active therapy boosted nurses’ empathy for patients, but their emotional engagement was put to the test when material conditions in mental institutions worsened from the mid-1930s until the 1950s. Cuts in funding during the economic recession and the devastating consequences of World War II put severe pressure on their working conditions.^50^ The suffering experienced by patients and nurses alike sometimes made them feel closer to each other, even though until the 1950s coercive measures were applied more often again, also on account of overpopulation and staffing shortages in many dilapidated mental institutions. In 1950, the state inspector reported on ‘negligence of the primary principles of humaneness’ and a ‘desperate mess of laziness and bigheadedness’ among the doctors in charge.^51^ Likewise, some nurses criticised the violent ways in which some of their colleagues, in part forced by the circumstances, applied coercion.^52^

In this period, several psychiatrists started to show more understanding for suicidal patients and to acknowledge that not every attempt could be prevented.^53^ Yet this did not automatically change the expectations regarding nurses. Only doctors were supposed to talk with patients about suicide, while the responsibility for its prevention and the effects of a suicide attempt were still resting on the shoulders of nurses. In 1938, for example, a psychiatrist argued that nurses should not engage at all in ‘philosophising’ on the underlying motives, for this might go at the expense of their attentiveness and close vigilance. It was their task ‘to do all they could to keep their patients from committing suicide’.^54^ At the same time, such prevention grew more difficult. A growing number of patients were hospitalised without court order and they insisted on a certain level of privacy and freedom. Constant monitoring was no longer feasible and instead the judgement of nurses grew more central: they were expected constantly to weigh ‘letting them move around freely’ and ‘giving trust’ on the one hand and ‘providing protection’ on the other.^55^ The risk of suicide and seeing someone try it from up close was a heavy burden on nurses, even though they were held accountable for it less often than before.^56^

In the area of sexuality, one can observe a cautious and tacit shift towards more pragmatism. Patients were still strictly separated based on gender and the nursing staff had to prevent masturbation and other sexual acts among patients. Sexual contacts between patients and nurses were met with heavy sanctions.^57^ In the denominational institutions, the dealing with sexuality continued to be more constrained than in the non-denominational ones, where nurses responded slightly less agitated and with more understanding to sexual desires and expressions by patients. Apparently, nurses were increasingly able to see that psychiatric patients, who because of their admission were often separated from their marital partner for lengthy periods of time, missed intimacy.^58^ Such empathy was stimulated by the fact that nurses themselves were gradually able to enjoy more liberties in the relationship sphere. Relations between male and female nurses no longer had to be silenced all the time and even homosexuality among colleagues was sometimes tacitly or discreetly tolerated, even though in patients this was still regarded as a disease or sexual offence which was sometimes treated medically (potentially involving castration).^59^

## The Regime of Personal Development

The growing government funding and regulation of mental health care from the 1950s onwards resulted in improvement of the material living and working conditions for patients and nurses.^60^ More and more mental patients received therapy instead of just being sheltered and cared for. Fences and closed doors began to be removed. The anonymity of large wards was replaced by care for smaller groups of patients, which made the interaction between nurses and patients more personal and more equal. Partly because of their new right to social security benefits, patients had more money to spend, which led to more means for personal care and facilitated the organisation of trips and vacations supervised by the nursing staff. Such improvements contributed to a further decrease of the emotional distance between both groups.

The introduction of psychotropic drugs, suppressing psychotic symptoms, brought optimism about the beneficial effects of socio- and therapeutic community treatment as well as creative and psychomotor therapy.^61^ While in previous periods the patients had to be educated towards self-responsibility with an eye to their social adaptation, now there was more emphasis on individual self-expression. Psychiatrists stressed that psychosocial therapies required the involvement of nurses who could systematically observe and report about patients’ behaviour, conduct group discussions and coach them, as well as listen to them carefully and understand them in an open-minded way. These innovations advanced the need for not only social skills, but also psychological insight among nurses, including ‘self-knowledge’ and ‘personality’ in order to be able to support ‘the creation of a therapeutic atmosphere’ aimed at the ‘positive encouragement’ of the mentally ill towards more autonomy. ^62^ Nurses were supposed to associate empathically with patients, while focussing on social supportive and therapeutic interaction, and thereby to reflect in a critical way on their own attitude. That nurses grew more empathic vis-à-vis the feelings of patients was reflected by, for instance, changes in bathing and personal care of patients. This used to take place collectively, whereby no attention was paid to privacy and feelings of shame among patients. Because this routine was more and more seen as embarrassing and humiliating, nurses began to provide personal care to patients individually, behind a closed bathroom door.^63^ Some of them began to raise doubts about occupational therapy: they viewed the monotonous work as humiliating, as serving no other purpose than to keep patients busy without therapeutic benefit. In general, nurses became more sensitive to the patients’ quality of life in mental institutions, whereas some of them also began to question the authority of psychiatrists and to express their own opinions about the treatment of patients. The first appeals for ending nurses’ subordination to doctors could be heard.^64^

The allergic reactions and apathy that psychotropic drugs induced in patients provoked criticism of nurses, but overall they applauded the new medication, in particular because other, more drastic somatic therapies, such as electroshock and mechanical restraint and isolation, which they experienced as painful, could be reduced.^65^ Drastic and labour-intensive coercive measures, such as wrapping and prolonged bath therapy, grew outdated, while straightjackets and similar forms were largely replaced by milder forms of restraint that were more bearable for patients and therefore also less troubling for nurses.^66^ Nurses also noted that having smaller units made it possible to calm down restless or aggressive patients through negotiation and patience.^67^ Still, some nurses saw their responsibility for applying coercion as burdensome, precisely because they empathised with their patients. This pertained not just to applying physical restraint, but also to administering high doses of psychiatric drugs.^68^

If coercive measures were applied markedly less or in less troublesome ways, the changes in dealing with suicide and sexuality were less drastic. Nurses still experienced their responsibility for suicidal patients as highly burdensome, also because in their training, apart from its emphasis on prevention and control, they hardly learned how to deal with the emotional aspects. If they could speak their minds about this at all, they did so with colleagues, not with doctors or inspectors.^69^

With few exceptions, mental institutions held on to a strict separation of the sexes way into the 1960s.^70^ On men’s wards, masturbation and homosexuality were often more noticeable than on women’s wards.^71^ The guidelines and instructions regarding sexuality were largely geared to its suppression. For example, nurses had to make sure that patients touched them and each other as little as possible. In this context, some considered the libido-reducing side effect of the psychopharmaceuticals rather as an advantage than as a problem.^72^ Sexuality was still hardly talked about openly. But according to some nurses, a more tacit understanding gradually emerged of the sexual needs of patients and even of intimate relations between a nurse and a patient.^73^

## The Emancipatory Regime

In the 1960s and 1970s, psychiatric nursing enjoyed great popularity among progressive youngsters in the Netherlands. Increasing numbers of better educated nurses were employed, as growing budgets for mental health care facilitated investments in psychiatric hospitals. The size of wards was further scaled down and the nurse–patient ratio halved between 1965 and 1975 from 1 to 4.5 to around 1 to 2.^74^ Psychiatric nurses were strongly affected by the critical attention in society for the fate of the mentally ill and the previously closed and isolated mental institutions opened up. The social–psychological and psychotherapeutic treatments, and the associated therapeutic optimism, now gained the upper hand and the participation of nurses in it intensified. Some of them joined the staff of therapeutic communities. Their interaction with doctors as well as patients became less hierarchical and more informal. Housekeeping duties that had traditionally been part of nursing, such as cleaning and meals, were transferred to domestic helps, and nurses started to exchange their white uniforms for more casual outfits. The strict rules that had applied to student nurses, such as mandatory institutional lodging, were relaxed. The use of the title *verpleegkundige*, referring to nursing as an expertise and current since the early 1960s, and the introduction of an ethical and disciplinary code, including confidentiality about patients, showed that nurses had gained professional status and also that they should respect the rights and dignity of patients.^75^

If many young psychiatric nurses were critical, they were also highly motivated. They organised themselves and voiced their views in the media, decrying not only ‘authoritarian’ psychiatrists, but also ‘old-fashioned’ nurses, who would show too little understanding and respect for patients. Such activism caused conflict with older colleagues who felt that the engagement of youngsters resulted in a neglect of their caring responsibilities for chronic patients, who were not amenable to the new psychotherapeutic ethos.^76^ Yet leading nurses increasingly participated in discussions about the redefinition of nursing in terms of guiding, stimulating and rehabilitating the mentally ill on the basis of open communication. The schooling of nurses should include pedagogy, psychology, psychotherapy and sociology, while learning social and communicative skills should reinforce the therapeutic effect of their daily interactions with patients. Training in reporting and writing was extended, in particular to engender a self-reflective attitude towards patients.^77^ Senior psychiatric nurses began to replace psychiatrists as instructors and examination supervisors, and in the 1970s, the dominance of physicians came to an end.^78^

In the course of the 1970s and 1980s, the emergence of the patients’ movement and the political–legal discussion on the admission procedures and coercion in psychiatry led to more protection of the rights of patients. It was no longer assumed that compulsory admitted patients had no full legal capacity and could be subjected to treatment without consent, ‘for their own good’.^79^ In 1979, the mandatory coercive means registration was further extended to include not only direct forms of physical restraint through fixation and separation, but also other restrictions in patients’ daily lives, such as lack of privacy and communication with the outside world. There was also attention for impermissible psychic and moral pressure. In this context, a government committee urged for ‘adhering to the proper ethical norms by those who deal with patients and who treat and care for patients, which implies, among other things, recognition of the individual’s personal dignity and refraining from an infantilizing approach’.^80^ After the appointment of independent mediators in the 1980s, whom patients could consult confidentially, nurses had to account more often for their dealing with patients, notably when involving the (still quite common) application of restraint and isolation.^81^ In the nursing curriculum, it was emphasised that self-reflection and self-control could reduce the use of ‘psychic coercive means’, and nurses themselves pushed for more training in the methods they could thereby use.^82^ Nurses should learn to react ‘more humanely’ to problematic conduct by patients by showing their emotions, while their training should pay more attention to dealing ‘with fear and risk’.^83^ Nurses should no longer have to do all they could to ensure self-control in all circumstances, and there should be room for them to express feelings vis-à-vis patients.^84^ Partly because of several notorious conflicts and legal cases, the debate on the right of nurses to refuse the use of coercive measures (pleading conscientious objections) grew stronger.^85^

The informalisation of forms of interaction in psychiatric hospitals was expressed in particular in the growing openness about sex and relations. In the 1970s, most mental institutions in the Netherlands introduced mixed male/female wards and soon it became clear that this led to a more relaxed atmosphere: male patients became less rude in their responses and women became interested again in their appearance. Also, because nurses were more often confronted with sexual needs and behaviours (including abuse) among patients, they grew more critical of the tabooisation and the lack of attention for it in their training.^86^ They developed a better sense of patients’ need for intimacy. In response to the introduction of mixed gender wards, a female nurse observed: ‘Patients also met each other in the evenings. This was unheard of in the past, when they were barely allowed to look at each other. […] And we saw that it was all-right […] The warmth patients expressed to each other, the comfort of just sitting together, doing things together, listening to each other’s trouble […]’.^87^

At the same time, however, new rules were formulated on the boundaries care providers themselves needed to respect. Patients received the right to self-determination and privacy.^88^ The more informal forms of interaction between nurses and patients and the improved rights of the latter brought along a greater sensitivity regarding power differences and possible boundary-transgressing behaviour. New, more relaxed norms on ‘professional detachment’ called for higher standards of self-control.^89^ The demands on their professional attitude and competences were substantial: they should show empathy towards patients without being too close, intrusive or over-concerned, as well as endure and channel patients’ possible problematic and offensive behaviour without displaying anger and frustration, while at the same time they should support them to navigate their daily life. The ensuing dilemmas presented themselves in particular in their dealing with suicidal patients.

In the 1970s, partly because of the growing recognition of the right to self-determination of patients, nurses were increasingly confronted with suicide.^90^ While more than previously they could count on a listening ear from their superiors and colleagues, patients who tried to commit suicide complained about the lack of understanding and emotional support by nurses (and psychiatrists).^91^ The fear of nurses for new suicide attempts often impeded an open dialogue on the events involved, which caused suicidal patients to exploit this fear as a means to put pressure on them or manipulate them.^92^ As the total social control of suicidal patients by nurses in the past was incompatible with their right to self-determination and privacy, the only option in threatening situations was to put such patients in isolation. Regardless of the unpleasant associations provoked by this rigorous coercive measure as such, the communication was complicated by the issue of their deprivation of freedom.^93^ Some argued in favour of setting a maximum term for isolation of suicidal patients, in which care providers should try to make them change their plan. If they were unsuccessful, however, lengthy isolation was not a solution. There was a limit to the coercion exercised to prevent suicide, but the determination of this limit was largely left to the judgement of the care providers involved, which continued their emotional burden.

## Conclusion

While in the Netherlands formal regulations of the nursing profession were mainly determined from above by others—mainly by asylum doctors and psychiatrists from the late nineteenth century to the mid-twentieth century, and afterwards increasingly by the government and vocational training institutions—nurses gradually gained more influence at the level of their daily work and thus they increasingly managed to shape their professional practice and identity. From the 1950s on psychiatrists in fact advanced this development by stressing the need for a self-reflective attitude of nurses. Power inequalities between doctors and nurses as well as between nurses and patients diminished, whereas norms and expectations of compassionate care and empathy grew more pronounced. The rules and expectations that guided how nurses should manage their feelings did not just depend on personal goodwill and virtues, but they were embedded within and mediated by wider institutional and socio-political factors. Together with improved material conditions, increasingly equal social relations contributed to more informal, flexible and negotiated interactions between nurses and patients. All of this affected the relationship deemed desirable between professional detachment—as first prescribed in rules imposed by superiors and later on in increasingly more formalised professional standards—and emotional commitment, whereby over time increasingly higher demands were placed on nurses in terms of self-reflection and self-regulation. Understanding and empathising with patients’ needs, preferences, viewpoints and feelings increasingly required reflexive thoughtfulness and emotional labour on the part of nurses.

Our finding confirms what the sociologist Cas Wouters, building on the theory of civilisation of Norbert Elias, put forward in a more general sense on processes of ‘formalisation’ and ‘informalisation’ in the twentieth century. Social democratisation in different sectors of society, he argues, came with a reduction of the power differences and social distance between people (with regard to class, sex, age and care dependency). This has been accompanied by refinement of the rules of behaviour, which allowed for more freedom and flexibility on the one hand, but which on the other hand require ever more subtle forms of emotional self-regulation.^94^ According to Wouters, such informalisation occurred some ten years earlier in the manners of people who professionally were much more exposed to intimate human relations and problematic behaviour (such as physicians and nurses) than others. We cannot fully confirm this last claim. Probably the changing attitude regarding deviant, painful and extreme patterns of behaviour in psychiatry did overall not essentially differ from that in other domains of society, although some psychiatrists were in the vanguard with regard to suicide and the mixing of male and female patients on wards.

What is more, the shift we observed from imposed do’s and don’ts towards more flexible negotiation patterns did not take place simultaneously, in a linear fashion or to same degree with respect to each of the three problem areas we studied. If the application of physical coercion evolved quite strongly, the changes pertaining to sexuality and suicide were less drastic and these were also realised later on and marked by more ambivalence. From the start of the profession’s formation, the reduction of physical coercion was a major motivation for setting up a nursing curriculum, initially largely for women. The changing way of dealing with sexuality and suicide, although noticeable, was much less explicitly discussed, also because these issues, in contrast to dealing with physical restraint, were harder to employ for the external portrayal of psychiatric nursing as a respectable profession, the more so because of the wider social tabooisation of suicide and sexuality. No one could be against the abolition of inhumane treatments, but openness about sexuality and suicide was a different matter, at least until the 1960s or 1970s, while also afterwards it continued to be ambiguous. A more pragmatic dealing with sexuality and suicide gradually and largely silently developed in the wake of the earlier changes in dealing with coercion. The application of physical restraints became a more painful issue for nurses after the introduction of active therapy caused a reduction of the emotional distance between nurses and patients. The improved living circumstances in mental institutions as of the 1950s and the growing attention for the self-expression of patients, which in the 1960s was followed by the introduction of mixed male/female wards, certainly resulted in a less rejecting attitude towards sexuality. The dealing with suicide changed mainly in the 1970s in response to the acquired right to self-determination of patients. Whereas initially the proximity of nurses vis-à-vis patients mainly had a physical component—as part of coercive measures and surveillance—and too much emotional involvement had to be suppressed, in the course of the twentieth century such empathy increased. At the same time, based on rights concerning bodily integrity and privacy, nurses had to approach patients with more physical distance and caution.

Our analysis ends in the 1980s, when neoliberal policies began to cut back on government funding and to introduce market mechanisms in health care. Psychiatry in general and mental nursing in particular have been affected by this shift, and we suspect that with respect to the balance between emotional distance and involvement a reverse trend prevailed in the past decades. The re-medicalisation of psychiatry, ongoing professionalisation inspired by nursing science, new technocratic managerial regimes, and the importance of budget considerations entailed that methodical and efficient operating procedures and accountability gained ground. As a consequence of more administrative responsibilities for nurses at a distance from the daily practice of care (‘office nursing’), nurses and patients complained that the emotional quality of care suffered because the social interactions between nurses and patients on the wards of mental hospitals became again more impersonal. Therefore, we suggest further historical analysis and examination of these recent developments with regard to the relation between professional detachment and emotional involvement. 

## Footnotes

1 H. van den Bor, ‘Iets over de verpleging van krankzinnigen’, *Maandblad van de Nederlandsche Verplegers Vakvereeniging*, 1916, 10, 153–58, 193–95, 213–19, 233–36, 235–36.

2 H. van den Bor, ‘Iets over de verpleging van krankzinnigen’, *Maandblad van de Nederlandsche Verplegers Vakvereeniging*, 1917, 11, 70–74, 144–50, 72–73.

3 E. Homburg, *Van beroep ‘Chemiker’. De opkomst van de industriële chemicus en het polytechnisch onderwijs in Duitsland (1790*–*1850)* (Delft: Delftse Universitaire Pers, 1993), 1–22; see also A. Abbott, *The System of Professions. An Essay on the Division of Expert Labor* (Chicago: The University of Chicago Press, 1988); A. J. G. van der Arend, *Beroepscodes. Morele kanttekeningen bij een professionaliseringsaspect van de verpleging* (Nijkerk: Intro, 1992), 21–57.

4 J. C. M. Hattinga Verschure, *Algemene zorgkunde voor de zorgverlenende beroepen* (Lochem, Poperinge: De Tijdstroom, 1981); P. Schnabel, ‘Professionaliteit als probleem in de geestelijke gezondheidszorg’, *Tijdschrift voor Sociale Gezondheidszorg*, 1984, 62, 602–08; J. Savage, ‘Relative Strangers: Caring for Patients as the Expression of Nurses’ Moral/Political Voice’, in T. Kohn and R. McKechnie, eds, *Extending the Boundaries of Care. Medical Ethics & Caring Practices* (Oxford, New York: Berg, 1999), 181–201; J. Pols, *Good Care. Enacting a Complex Ideal in Long-Term Psychiatry* (Utrecht: Trimbos-instituut, 2004).

5 A. R. Hochschild, ‘Emotion Work, Feeling Rules, and Social Structure’, *American Journal of* Sociology, 1979, 85, 551–75.

6 Archief Inspecteurs van het Staatstoezicht op Krankzinnigen en Krankzinnigengestichten, Nationaal Archief (The Hague). Praktijk- en rapportenboekjes (1924–1970), inventory 2.15.40.

7 Essays by mental nurses submitted in 1941 writing contest, Archief Examen Commissie van de Nederlandse Vereniging voor Psychiatrie en Neurologie, Noord-Hollands Archief (Haarlem), inventory 6.

8 Completed questionnaires, anonymous respondents, 2006–2007. Personal archive C. aan de Stegge.

9 Authorized interviews, Personal archive C. aan de Stegge.

10 J. van Deventer, W. P. Ruysch and A. O. H. Tellegen, ‘De verbetering van het verplegend personeel in de Nederlandsche Krankzinnigengestichten’, *Psychiatrische Bladen*, 1890, 8, 222–36; J. Stuten-te Gempt, *Mijne ervaringen in het krankzinnigengesticht te ‘s-Gravenhage, Slijkeinde no. 2* (‘s-Gravenhage: J. Hoekstra, 1892); E. J. A. Melster, *Mishandelingen ondervonden in het ‘Oude en Nieuwe Gasthuis, Geneeskundig Gesticht voor Krankzinnigen’ te Zutfen* (Enschede: M. J. van der Loeff, 1902).

11 N. B. Donkersloot, *Raadgevingen aan oppassers en bedienden in krankzinnigengestichten* (Dordrecht: Geneeskundig gesticht voor Krankzinnigen, 1865); F. A. Hartsen, *De Handleiding voor Oppassers in Gasthuizen voor Krankzinnigen van J. Curwen M. D., voor Nederland gewijzigd* (Utrecht: Kemink & Zn. Hartsen, 1862); P. van der Esch, *Geschiedenis van het Staatstoezicht op Krankzinnigen*, 3 vols (Leidschendam: Ministerie van Volksgezondheid en Milieuhygiëne, 1975–1980), I, 42–49.

12 H. Oosterhuis, H. and M. F. Gijswijt-Hofstra, *Verward van geest en ander ongerief. Psychiatrie en geestelijke gezondheidszorg in Nederland (1870–2005)* (Houten: Bohn, Stafleu/Van Loghum, 2008), 70–185.

13 W. H. Cox, ‘De verpleegsters-kwestie’, *Psychiatrische en Neurologische Bladen*, 1898, 2, 234–42, 234, 242.

14 A. Etzioni, ed, *The Semi-professions and Their Organization. Teachers, Nurses, Social Workers* (New York: The Free Press, 1969); C. Davies, *Gender and the Professional Predicament in Nursing* (Buckingham, Philadelphia: Open University Press, 1995).

15 J. van Deventer, ‘Eenige opmerkingen over de psychische behandeling van krankzinnigen’, *Psychiatrische Bladen*, 1888, 6, 20–31; J. van Deventer, ‘Eenige beschouwingen over krankzinnigenverpleging’, *Psychiatrische Bladen*, 1892, 10, 232–51; Van Deventer, Ruysch and Tellegen, ‘De verbetering van het verplegend personeel’; J. van Deventer, *Handboek der Krankzinnigenverpleging* (Amsterdam: J. H. & G. van Heteren, 1897); B. van Delden, *Onze krankzinnigen en hunne verpleging* (Utrecht: C.H.E. Breijer, 1897); J. C. Th. Scheffer, *Voorlezingen over zenuwzieken en krankzinnigen en hunne verpleging* (Haarlem: De Erven F. Bohn, 1906 and 1914); P. H. M. Travaglino, *Leidraad bij de voorbereiding tot het examen in krankzinnigenverpleging* (Amsterdam: F. van Rossen, 1910); J. G. Schnitzler, *Krankzinnigen en hun verpleging* (Amsterdam: J.H. De Bussy, 1915); P. J. M. Aalberse and Ch. Ruijs de Beerenbrouck, ‘Wet tot wettelijke bescherming van het diploma voor ziekenverpleging. Besluit 702’, *Staatsblad*, 1921, 21 May 253.

16 G. J. C. aan de Stegge, *Gekkenwerk. De ontwikkeling van het beroep psychiatrisch verpleegkundige in Nederland 1830-1980* (Maastricht: Universitaire Pers Maastricht, 2012), 222–23.

17 G. Boschma, *The Rise of Mental Health Nursing. A History of Psychiatric Care in Dutch Asylums 1890-1920* (Amsterdam: Amsterdam University Press, 2003), 188, 199–204; Aan de Stegge, *Gekkenwerk*, 240–41, 303, 1010; E. Borgesius, J. de Lange and P. Meurs, *Verpleegkundigen zonder uniform, over de pluriformiteit van het beroep psychiatrisch verpleegkundige* (Utrecht: NcGv/De Tijdstroom, 1988), 191.

18 Van Delden, *Onze krankzinnigen en hunne verpleging*, 171; Van Deventer, *Handboek der Krankzinnigenverpleging*, 53; D. Schermers, *Handleiding bij het verplegen van krankzinnigen* (Leiden: D. Donner, 1898), 127, 222.

19 Schermers, *Handleiding bij het verplegen van krankzinnigen*, 188; Van Delden, *Onze krankzinnigen en hunne verpleging*, 75–85; Scheffer, *Voorlezingen over zenuwzieken en krankzinnigen*, 221–24.

20 W. H. Cox et al., ‘Inlichtingen en Wenken voor de examens, af te nemen vanweg de Nederlandsche Vereeniging voor Psychiatrie en Neurologie’, *Psychiatrische en Neurologische Bladen*, 1904, 8, 810–39, 837.

21 C. aan de Stegge, ‘Changing Attitudes Towards “Non-Restraint” in Dutch Psychiatric Nursing, 1897-1994’, in M. Gijswijt-Hofstra et al., eds, *Psychiatric Cultures Compared. Psychiatry and Mental Health Care in the Twentieth Century: Comparisons and Approaches* (Amsterdam: Amsterdam University Press, 2005), 331–58.

22 Van Delden, *Onze krankzinnigen en hunne verpleging*, 195.

23 F. Koetser and L. Marseille, eds, *Dwangmiddelenregistratie: legitimatie of willekeur? Verslag van de studiedag op 13 januari 1983* (Amsterdam: Stichting Pandora, 1983), 20, appendix: document 10; D. Schermers, *Handleiding bij het verplegen van Krankzinnigen en Zenuwlijders* (Leiden: Gebr. Van der Hoek, 1911), 353.

24 J. Vijselaar, *Het gesticht, enkele reis of retour* (Amsterdam: Boom, 2010), 215–22.

25 W. M. van der Scheer, *Nieuwere inzichten in de behandeling van geesteszieken* (Groningen, Den Haag, Batavia: Wolters, 1933), 30.

26 Inspecteurs voor het Staatstoezicht op de Krankzinnigen en de Krankzinnigengestichten in Nederland, ‘Verslag van het Staatstoezicht op Krankzinnigen over 1888’, *Psychiatrische Bladen*, 1889, 7, 210–58, 225; J. Vijselaar, *Krankzinnigen gesticht. Psychiatrische inrichtingen in Nederland, 1880-1910* (Utrecht: NcGv, 1992), 109.

27 A. M. Geurts, ‘Twintig jaren’, 1941 writing contest, 1**–**2.

28 J. Kat, ‘Reorganisatie van het verplegend personeel in de Rijkskrankzinnigengestichten’, *Psychiatrische en Neurologische Bladen*, 1904, 8, 73**–**83, 81.

29 Hartsen, *De Handleiding voor Oppassers*, 46; Van Deventer, *Handboek der Krankzinnigenverpleging*, 12–17, 77, 93; Van Delden, *Onze krankzinnigen en hunne verpleging*, 146; Schermers, *Handleiding bij het verplegen van krankzinnigen*, 207.

30 Vijselaar, *Het gesticht, enkele reis of retour*, 215.

31 Van Deventer, *Handboek der Krankzinnigenverpleging*, 58–60; Van Delden, *Onze krankzinnigen en hunne verpleging*, 25–33, 138–39, 165–68; Schermers, *Handleiding bij het verplegen van krankzinnigen*, 228–30; Scheffer, *Voorlezingen over zenuwzieken en krankzinnigen*,175–79; Boschma, *The Rise of Mental Health Nursing*, 133–35; J. de Roos, ‘Hora ruit’, 1941 writing contest, 4; D. van der Horst, ‘Het is een groot voorrecht gezond te zijn’, 1941 writing contest.

32 Schermers, *Handleiding bij het verplegen van krankzinnigen*, 228–30.

33 Z. T. Blankenstein, ‘Het belang van de ons toevertrouwde zieken komt altijd op de eerste plaats’, 1941 writing contest, 4.

34 A. van der Brug, ‘Nog steeds bergopwaarts’, 1941 writing contest, 28; Geurts, ‘Twintig jaren’, 18; Van der Horst, ‘Het is een groot voorrecht gezond te zijn’; Van Deventer, *Handboek der Krankzinnigenverpleging*, 61, 65; Van Delden, *Onze krankzinnigen en hunne verpleging*, 165; Schermers, *Handleiding bij het verplegen van krankzinnigen*, 230.

35 F. Dommisse, *Krankzinnigen* (Rotterdam: W. L. & J. Brusse’s Uitgeversmaatschappij, 1929), 30; Blankenstein, ‘Het belang van de ons toevertrouwde zieken’, 19; Geurts, ‘Twintig jaren’, 18.

36 A. Stärcke, ‘Moderne behandeling van geesteszieken’, *Nederlandsch Maandschrift voor Geneeskunde*, 1931, 17, 313–67; Van der Scheer, *Nieuwere inzichten in de behandeling van geesteszieken*; F.S. Meijers, ‘Beschouwingen van een buitenstaander over onze moderne gestichtsverpleging’, *Psychiatrische en Neurologische Bladen*, 1936, 40, 221–29, 228; Aan de Stegge, *Gekkenwerk*, 421–42.

37 Oosterhuis and Gijswijt-Hofstra, *Verward van geest en ander ongerief*, 270–73; C.Th. Bakker, *Geld voor GGz. De financiering van de geestelijke gezondheidszorg en de invloed van geld op de zorgpraktijk (1884-1984)* (Amsterdam: Amsterdam University Press, 2009), 39–40.

38 Aan de Stegge, *Gekkenwerk*, 188–92.

39 F. Dommisse, *Opdat er kieme.…, beschouwingen over gestichtsverpleging van geesteszieken* (Rotterdam: W. L. & J. Brusses’ Uitgeversmaatschappij, 1939); J. Clay, ‘Geduld en toewijding’, 1941 writing contest; Geurts, ‘Twintig jaren’; J. Mertz, ‘Uit het duister tot het licht’, 1941 writing contest.

40 Geurts, ‘Twintig jaren’, 2, 12–14; C. de Bruijn, ‘Willen is Kunnen’, 1941 writing contest; J. H. H. Versteeg, ‘Muziek en verpleging’, 1941 writing contest.

41 Geurts, ‘Twintig jaren’ , 12–13; Clay, ‘Geduld en toewijding’.

42 ‘Koninklijk Besluit’, *Staatsblad*, 1930, *October* 13, 404. Koetser and Marseille, *Dwangmiddelenregistratie: legitimatie of willekeur?*, appendix: document 11.

43 Letter Piet Rooijackers to Cecile aan de Stegge, May 21, 2002, 2005, 1, Personal archive Aan de Stegge; A. Kaptijn, *Herinneringen bij het ouder worden* (Urk: unpublished manuscript, ca. 1990), 22, 36; Completed questionnaire Van Ginkel-Van Runt, 3; Clay, ‘Geduld en toewijding’.

44 R. Osinga, ‘Blijf vóór. Arbeid in onze gestichten’, 1941 writing contest, 4.

45 Authorized interview, Jan de Haan, 14 September 1999.

46 C. A. Dardenne Ankringa, ‘Eerbied voor het leven’, *Tijdschrift voor Ziekenverpleging*, 1942, 52, 116–18; 129–34; 152–55, 52, 116–18, 129–34, 152–55.

47 Dardenne Andringa, ‘Eerbied voor het leven’, 55–73, 149–59.

48 Clay, ‘Geduld en toewijding’, 17.

49 F. Boon, **‘**In depressie**’,** 1941 writing contest.

50 Oosterhuis and Gijswijt-Hofstra, *Verward van geest en ander ongerief*, 437, 465–513.

51 Letter J. H. Pameijer to Prof. Dr G. Kraus, May 11, 1950, 2. Personal archive Aan de Stegge.

52 Completed questionnaire HS, 1, 3; Letter Herbert Scherpenzeel to Cecile aan de Stegge, June 1, 2002. Personal archive Aan de Stegge.

53 J. H. Haverkate and B. Chr. Hamer, *Schermers’ Leerboek bij het verplegen van krankzinnigen en zenuwzieken* (Leiden: Gebr. Van der Hoek, 1946), 421; J. H. Haverkate and B. Chr. Hamer, *Schermers’ Leerboek bij het verplegen van geestes- en zenuwzieken* (Amsterdam: Uitgeversmaatschappij van Mantgem & De Does, 1950), 432.

54 H. C. Jelgersma, ‘De bestrijding van den zelfmoord in psychiatrische inrichtingen’, *Tijdschrift voor Ziekenverpleging*, 1938, 48, 416–21; 440–47; 468–73; 498–503, 416–17.

55 T. Duursma, ‘In dienst voor de mensheid’, 1941 writing contest, 6; Jelgersma, ‘De bestrijding van den zelfmoord in psychiatrische inrichtingen’.

56 G. C. Moleman, ‘Tracht door liefde lijden te verzachten’, 1941 writing contest, 15–16.

57 Authorized interview, Philip Kemp, March 10, 2004, 9–10.

58 H. J. Schim van der Loeff and J. A. J. Barnhoorn, *Zielszieken en hunne Verpleging* (Roermond: J. J. Romen & Zonen, 1930), 284; H. J. Schim van der Loeff and J. A. J.Barnhoorn, *Zielszieken, zenuwzieken en hun verpleging* (Roermond: J. J. Romen & Zonen, 1936), 355; H. J. Schim van der Loeff and J. A. J. Barnhoorn, *Zielszieken, zenuwzieken en hun verpleging* (Roermond: J. J. Romen & Zonen, 1947), 365; Haverkate and Hamer, *Schermers’ Leerboek bij het verplegen van krankzinnigen en zenuwzieken* (1932), 340; (1938), 362–63; (1946), 421–22; (1950), 326–27, 433; De Roos, ‘Hora ruit’, 8–9; Authorized interview Kemp, 9–10; Completed questionnaire JS, 3; Completed questionnaire AS, 3; Letter Rooijackers, 1.

59 P. C. Kuiper, *Ver heen* (’s Gravenhage: SDU, 1988), 150**–**51; Authorized interview Kemp, 10**–**11; Completed questionnaire HS; Completed questionnaire JvD, 3.

60 Aan de Stegge, *Gekkenwerk*, 643–47.

61 Oosterhuis and Gijswijt-Hofstra, *Verward van geest en ander ongerief*, 533–75; F. Meyboom, ‘De taak van de verplegenden in een psychiatrische inrichting’, *Maandblad voor de Geestelijke Volksgezondheid*, 1952, 7, 25–29.

62 A. P. Timmer, *Leerboek voor verplegenden van zenuwzieken en krankzinnigen* (Haarlem: De Erven F. Bohn, 1952), 322; (1957), 341–42, 345, 347; see also B. Chr. Hamer and F. J. Tolsma, *Algemeen Leerboek voor het verplegen van geestes- en* zenuwzieken (Leiden: Spruyt, Van Mantgem & De Does N.V., 1956), 438–39, 595–96.

63 Completed questionnaire KM.

64 P. Stevens, J. Pepping and A. P. Lammens, *Psychiatrische Verpleegkunde*. 3 vols. (Heiloo: Stichting St. Willibrord, 1960-1963).

65 Aan de Stegge, *Gekkenwerk*, 771–72, 794, 802, 609–16.

66 Authorized interview De Haan ; Letter Piet Rooijackers to Cecile aan de Stegge, July 27, 2005, 2, Personal archive Aan de Stegge.

67 H. van der Drift, *Gezag, tucht, inrichtingsdiscipline in het kader van de heropvoeding* (Interne Publicatie Wolfheze, 1952),11; Authorized interview, Jan Klungers, March 13, 2002; Authorized interiew, De Haan.

68 Completed questionnaire PR; Completed questionnaire GvdB; Completed questionnaire GL; Completed questionnaire AJ; Completed questionnaire JdL; Completed questionnaire JV; Completed questionnaire HvdW; Completed questionnaire GH; Authorized interview, Joke Zwanikken-Leenders, August, 21 2002.

69 Authorized interview, Zwanikken-Leenders; Completed questionnaire JS; Completed questionnaire AD; Completed questionnaire GV; Completed questionnaire GvdB; Completed questionnaire HvdW; Completed questionnaire PS; Completed questionnaire LJ; Completed questionnaire CR; Completed questionnaire IvdP; Completed questionnaire WW; Completed questionnaire HL; Completed questionnaire JZ-L.

70 Authorized interview, Dicky van Dronkelaar-Otterman, 7 March 2003.

71 Completed questionnaire HL; Completed questionnaire KM.

72 Completed questionnaire Lettink.

73 Completed questionnaire AJ; Completed questionnaire JdL; Completed questionnaire PR; Completed questionnaire RvE; Completed questionnaire JS; Completed questionnaire ML-vW; Completed questionnaire JK-K; Completed questionnaire MW, 3–4; Authorized interview, Willy van Driel, September 11, 2006.

74 A. J. Gerritse and W. van der Windt, *Van verloop naar loopbaan, een arbeidsmarktverkenning verpleging en verzorging in de negentiger jaren.* Rapport nr. 91.724 (Utrecht: Nationaal Ziekenhuisinstituut, 1991), 87; P. C. J. van Loon, ‘Eindrapport van de Commissie Hogere Beroepsopleiding Verplegenden’, *Verslagen en Mededelingen Volksgezondheid*, 1969, 24, 9–46; Nationale Ziekenhuisraad, *De arbeidsmarkt voor inservice-leerlingen, een onderzoek naar de stand van zaken en toekomstige ontwikkelingen* (Utrecht: Nationale Ziekenhuisraad, 1985), 6.

75 Werkgroep Rechtspositie Patiënten in Psychiatrische Ziekenhuizen, *Resultaten enquête* (‘s-Gravenhage: Ministerie van Volksgezondheid en Milieuhygiëne, 1979); Werkgroep Rechtspositie Patiënten in Psychiatrische Ziekenhuizen, *Eindrapport* (‘s-Gravenhage: Ministerie van Volksgezondheid en Milieuhygiëne, 1979).

76 Authorized interview, Evert Verkerk, February 10, 2004; Authorized interview, Marianne van de Siepkamp-de Vries, 26 February 2004; Authorized interview, Theo van der Donk, 3 June 2004.

77 W. A van den Hurk, *Bewaken wordt begeleiden, moderne psychiatrische verpleging* (Amsterdam: IVIO, AO reeks, 1966); Van Loon, Eindrapport van de Commissie Hogere Beroepsopleiding Verplegenden; C. J. M. Nieland, L. van der Laan and P. F. Rooyackers, *Verpleegkundige lessen voor de opleiding tot het diploma B* (Leiden: Spruyt, Van Mantgem & De Does, 1969); J. A. M. Kerstens, *Grondkaders voor een leerplan psychiatrische verpleegkunde* (Lochem: De Tijdstroom, 1974).

78 F. Kramer, *Psychiatrische verpleegkunde* (Lochem: De Tijdstroom, 1968); Nieland, Van der Laan and Rooyackers, *Verpleegkundige lessen voor de opleiding tot het diploma B*.

79 P. J. H. Laurs, *Recht voor psychiatrische patiënten. Een onderzoek naar de rechtsbescherming van in psychiatrische ziekenhuizen opgenomen patiënten* (Arnhem: Gouda Quint, 1988), 47**–**49; C. J. van de Klippe, *Dwangtoepassing na onvrijwillige opname. Een juridische beschouwing* (Nijmegen: Ars Aequi Libri, 1997), 43, 46, 48**–**49; P. Bierenbroodspot, *De therapeutische gemeenschap en het traditionele psychiatrische ziekenhuis* (Meppel: Boom, 1969); C. van Eijk-Osterholt, *Laten ze het maar voelen….Vijf en twintig jaar belangenbehartiging voor een verpleegde* (Amsterdam: Van Gennep, 1972 and 1981).

80 Koetser and Marseille, *Dwangmiddelenregistratie: legitimatie of willekeur?*, 31–33; Werkgroep Rechtspositie Patiënten in Psychiatrische Ziekenhuizen, *Eindrapport*, 18, 25, 40.

81 P. J. H. Laurs, ‘Naar een stichting “Ombudswerk in de Psychiatrie”?’, *Tijdschrift voor Gezondheidsrecht*, 1979, 3, 228**–**45; P. J. H. Laurs, ‘Van ombudsman tot patiëntenvertrouwenspersoon. Ontwikkelingen en perspectieven van de klachtenbehandeling in de geestelijke gezondheidszorg’, *Maandblad Geestelijke Volksgezondheid*, 1982, 42, 750**–**63, 761; A. Frid, P. Ippel and P. Laurs, *Jij liever dan ik. De psychiatrische patiënt, wat heeft hij te vertellen?* (’s Gravenhage: VUGA Boekerij, 1980), 82; J. Legemaate, *Afzondering. Over celisolatie in de psychiatrie.* Unpublished Master Thesis (Utrecht: Willem Pompe Instituut voor Strafrechtswetenschappen, 1982), 18, 95.

82 Frid, Ippel en Laurs, *Jij liever dan ik* 1980, 31**–**32; C. Bennink, ‘Inleiding’, in Werkgroep Kwaliteit en Doelmatigheid van de Subsectie Psychiatrie van de Nationale Ziekenhuisraad, *Studiedag ‘Tegen je zin’, gehouden op 26 mei 1983 in de Lucaskerk van het Psychiatrisch Ziekenhuis Veldwijk te Ermelo* (Utrecht: NZR, 1983), 5**–**11, 10**–**11; Koetser and Marseille, *Dwangmiddelenregistratie: legitimatie of willekeur?*, 19**–**20; G. Houwink, ‘Inleiding’, in Werkgroep Kwaliteit en Doelmatigheid van de Subsectie Psychiatrie van de Nationale Ziekenhuisraad, *Studiedag ‘Tegen je zin’*.

83 H. van de Klippe, ‘De B-verpleegkundige onder de BOPZ. Verslag van Studiedag Het Beterschap 29-5-1984’, *Maandblad Geestelijke Volksgezondheid*, 1984, 39, 1210–14, 1212.

84 R. W. Piepenbrink, ‘Machtspatronen in de psychiatrische verpleegkunde’, *Maandblad Geestelijke Volksgezondheid*, 1986, 41, 979–91.

85 Van de Klippe, De B-verpleegkundige onder de BOPZ, 1213–14; Legemaate, *Afzondering*, 44; Bennink, ‘Inleiding’.

86 Completed questionnaire JN; Completed questionnaire HG; Completed questionnaire GvdW; Completed questionnaire AO; Completed questionnaire LJ; Completed questionnaire JK; Completed questionnaire CR; Completed questionnaire WL; Completed questionnaire HG; Completed questionnaire JvD; Completed questionnaire WW; Authorized interview, Zwanikken–Leenders; Authorized interview, Kemp, 11; Authorized interview, Klungers; Authorized interview, Jan Nijhof, 26 April 2011; U. van der Aa, *Maskers en machten. Vergeten psychiatrie* (Halsteren: Lucie Kerstens, 2008), 162–63; M. van Schayk, *Hallo medemens! De geschiedenis van het Werkteater* (Amsterdam: Theater Instituut Nederland, 2001), 60–61.

87 L. Nieuwenhuis and H. Te Brake, ‘”Eerst zien, dan geloven”. Interview met Riet Verschoor’, *Nieuwsbrief van de Vereniging Werkers in Psychotherapeutische Gemeenschappen*, 1991, 20, 4–7.

88 R. Offerhaus, *Wachten op oneindig. De chronische patiënt in de psychiatrische inrichting* (Deventer: Van Loghum Slaterus, 1983), 173; Werkgroep Kwaliteit en Doelmatigheid Psychiatrische Ziekenhuizen, *Handleiding voor discussie* (Utrecht: Nationale Ziekenhuisraad, Sectie Geestelijke Gezondheidszorg, 1979), 4.

89 H. J. Dalewijk, ‘De juridische aspecten: de kwetsbare positie van de medewerker’ in *Verslag van de studiemiddag (on)gewenste intimiteit en sexualiteit in de relatie tussen patiënten en medewerkers, 16 juni 1988*, *Psychiatrisch Centrum Zon en Schild* (Amersfoort: Zon en Schild, 1988), 5–10; W. Wesselink, ‘Kwaliteit in intiem professioneel kontakt’, in Ibid., 17–20; J. B. van Borssum Waalkes, *Bondgenoot van de zwaksten. Hulpverleners in het psychiatrisch ziekenhuis* (Amsterdam: Balans, 1991), 111–25.

90 A. Kerkhof, *Suïcide en de geestelijke gezondheidszorg* (Lisse: Swets en Zeitlinger, 1985), 13, 37, 44; A. C. de Graaf, ‘Zelfmoord in psychiatrische ziekenhuizen’, *Tijdschrift voor Psychiatrie*, 1976, 18, 814**–**21; A. C. de Graaf, ‘Zelfdodingen van opgenomen patiënten in en buiten psychiatrische ziekenhuizen in de periode 1970-1977. Mededelingen uit het Centraal patiëntenregister’, *Tijdschrift voor Psychiatrie*, 1982, 24, 506**–**12, 508, 511; F. van Ree, ‘Enkele gegevens uit een exploratieve studie naar suïcide in een psychiatrisch Ziekenhuis’, *Tijdschrift voor Psychiatrie*, 1981, 23, 382**–**98, 391; Offerhaus, *Wachten op oneindig*, 129**–**39.

91 Completed questionnaire PS; Completed questionnaire AH; Completed questionnaire JK.

92 Authorized interview, Flip Willemsen, June 8, 2001; Kerkhof, *Suïcide en de geestelijke gezondheidszorg*, 149, 152**–**53, 161.

93 Geneeskundige Inspektie voor de Geestelijke Volksgezondheid, *Referentiekader Middelen en Maatregelen. Derde versie, november 1987* (Leidschendam: Geneeskundige Inspektie voor de Geestelijke Volksgezondheid, 1987), 20, 23, 25; A. Poot, ‘75 jaar B-verpleegkunde, Episoden uit de Valeriuskliniek te Amsterdam’, *Maandblad Geestelijke Volksgezondheid*, 1985, 40, 1297–1313, 1312.

94 C. Wouters, *Informalisation. Manners and Emotions since 1890* (London: Sage, 2007).

